# Engraftment of Human Primary Acute Myeloid Leukemia Defined by Integrated Genetic Profiling in NOD/SCID/IL2rγnull Mice for Preclinical Ceramide-Based Therapeutic Evaluation

**DOI:** 10.4172/2329-6917.1000146

**Published:** 2014-07-25

**Authors:** Brian M Barth, Nichole R Keasey, Xujung Wang, Sriram S Shanmugavelandy, Raajit Rampal, Todd Hricik, Myles C Cabot, Mark Kester, Hong-Gang Wang, Leonard D Shultz, Martin S Tallman, Ross L Levine, Thomas P Loughran, David F Claxton

**Affiliations:** 1 Department of Medicine, Penn State College of Medicine, Hershey, USA; 2 Penn State Hershey Cancer Institute, Penn State College of Medicine, Hershey, USA; 3 Department of Pharmacology, Penn State College of Medicine, Hershey, USA; 4 Leukemia Service, Department of Medicine, Memorial Sloan-Kettering Cancer Center, USA; 5 Human Oncology and Pathogenesis Program, Memorial Sloan-Kettering Cancer Center, USA; 6 Department of Biochemistry and Molecular Biology, East Carolina University, Greenville, USA; 7 University of Virginia Cancer Center, Charlottesville, USA; 8 Department of Pediatrics, Penn State College of Medicine, Hershey, USA; 9 The Jackson Laboratory, Bar Harbor, USA

**Keywords:** Acute myeloid leukemia, Integrated genetic profiling, DNMT3A, NSG mouse, Ceramide, Tamoxifen, Nanoliposome

## Abstract

Acute Myeloid Leukemia (AML) is a highly heterogeneous and poor prognosis disease with few available therapeutic options. Novel advances are urgently needed, however effective models to test experimental therapeutics have been lacking. Recently, NOD/SCID/IL2rγnull (NSG) mice were shown to engraft primary human AML in a manner that recapitulated the natural disease and its progression. Additionally, integrated genomic profiling was used to refine risk stratification of AML. In this study, we demonstrated the engraftment of molecularly defined primary AML in NSG mice. We showed that AML that express DNMT3A mutations, which predict for adverse outcome, engrafted with exceptional efficacy. Lastly, we demonstrated that human AML-engrafted NSG mice can be effectively used to study novel ceramide-based therapeutics. Ceramide is a bioactive sphingolipid that has been implicated as an inducer of apoptosis. Elevation in cancer cell ceramide levels either via exogenous delivery or by provoking intracellular ceramide generation is the goal of ceramide-based therapeutics. In this study, we used the human AML-engrafted NSG mouse model to evaluate nanoliposomal short-chain C6-ceramide and a nanoliposomal formulation of the ceramide-inducer tamoxifen. Altogether, the NSG model is likely to prove invaluable in the study of novel agents, sushc as ceramide-based therapeutics, with the ability to define therapeutic activity against specific molecularly defined and risk stratified AML.

## Introduction

Acute Myeloid Leukemia (AML) is a growing public health problem in the United States with few available therapeutic options. AML is a highly heterogeneous disease or group of diseases. The 2008 WHO classification is accepted for clinical AML classification and incorporates known karyotypic data of prognostic importance but fails to predict outcome for many cytogenetically normal AML patients [1,2]. Recently, specific mutations were identified that predict AML outcome and improve risk stratification independent of historically recognized risk factors [2,3]. We had previously shown that this schema included mutations in TET2, DNMT3A, ASXL1, PHF6, and MLL, which predicted for adverse outcome, as well as patients with coincident mutations in NPM1 and in IDH1/2, which were associated with favorable outcome [2].

Novel advances for the treatment of AML are urgently needed, however appropriate models to test experimental therapeutics have been lacking. The translation of laboratory studies to clinical studies is inherently dependent on the usefulness of preclinical animal models. In the case of AML, murine models have proved disappointing for therapeutic development due primarily to the inability to replicate the human disease. The engraftment of human AML stem cells within the bone marrow of sublethal-irradiated NOD/SCID/IL2rγnull (NSG) mice has recently been reported, with these mice developing a more efficient disease compared with other immunodeficient strains [4–9]. Of particular note, enhanced engraftment of human AML with FLT3 mutations was reported as compared with wild-type FLT3-containing human AML [7]. Improvements on engraftment efficiency within this model have focused on transgenic expression of genes expressing human cytokines and growth factors [10]. Unfortunately, reports of the utility of this model for novel therapeutic testing are limited [6,11,12]. In the present report, we have used integrated mutational profiling to refine the risk stratification of human AML samples which successfully engrafted into NSG mice. We describe robust engraftment efficiency, with the novel finding that cases harboring DNMT3A mutations engrafted with exceptional efficacy. Finally, we provide proof of concept that the NSG model can be used to study the anti-AML efficacy of nanoliposomal C6-ceramide and nanoliposomal tamoxifen, which are novel ceramide-based therapeutics developed by our laboratories [13,14].

## Materials and Methods

### Integrated Mutational Profiling

Human AML samples were collected with informed consent and approval from the Penn State College of Medicine Institutional Review Board. Mononuclear cells were prepared by Ficoll separation, and cryopreserved prior to use. Genomic DNA was collected by extraction using a Qiagen DNeasy Kit according to the manufacture’s recommendations. Mutational profiling was performed as previously described in detail [2]. Briefly, mutational analysis of the entire coding regions of TET2, ASXL1, DNMT3A, PHF6, WT1, TP53, EZH2, RUNX1, and PTEN and of coding exons of FLT3, HRAS, KRAS, NRAS, KIT, IDH1, and IDH2 with known somatic mutations was performed using PCR amplification with the RainDance multiplex microdroplet PCR algorithm followed by Illumina HiSeq sequencing to ensure high coverage in the entire target region.

### Nanoliposome Preparation

Nanoliposomes were prepared as previously described [14–21]. Briefly, lipids dissolved in chloroform, were combined in specific molar ratios, dried to a film under a stream of nitrogen, and then hydrated by addition of 0.9% NaCl. Solutions were sealed, heated at 60°C for 60 minutes, and subjected to vortex mixing and sonicated until light no longer diffracted through the suspensions. The lipid vesicle-containing solutions were quickly extruded at 60°C by passing the solutions 10 times through 100 nm polycarbonate filters using an Avanti Mini-Extruder (Avanti Polar Lipids, Alabaster, AL). Nanoliposomal size and a neutral charge were validated using a Malvern Zetasizer Nano ZS at 25°C. Nanoliposome solutions were stored at room temperature until use.

### NSG Engraftment and Treatment

Briefly, adult NOD.Cg-PrkdcscidIl2rgtm1Wjl/SzJ (NSG) mice (8–10 wks old) received 250 cGy of sublethal total body irradiation, followed by tail vein injection of primary human AML cells. Cryopreserved cells were injected intravenously at doses of 0.5–10×106 viable mononuclear cells containing 80–90% leukemic blasts. Engraftment was monitored in by flow cytometry in 20 μl samples of tail vein peripheral blood with anti-human CD13, CD33, HLA-DR, and CD45 antibodies at 1–4 week intervals. Engraftment was defined as >0.5% cells expressing human CD45 and one other human marker. For subsequent transplantation, mice were euthanized and bone marrow mononuclear cells were harvested from tibiae and femurs and injected into recipients. For therapeutic evaluation, C6-ceramide nanoliposomes or tamoxifen nanoliposomes were administered via tail vein injection as previously described three times per week for up to two months [14–20]. Treatments were commenced following verification of engraftment in peripheral blood (>0.5%), and human AML burden was routinely monitored in the peripheral blood. Specifically, 20 μl blood collected by pricking the tail vein was added in precise volume to a solution of antibodies and counting beads. Blood was subsequently evaluated by flow cytometry using either a BD Canto II or BD LSR II flow cytometer. The Penn State College of Medicine Institutional Animal Care and Use Committee approved all animal procedures.

## Results and Discussion

**Figure 1:**
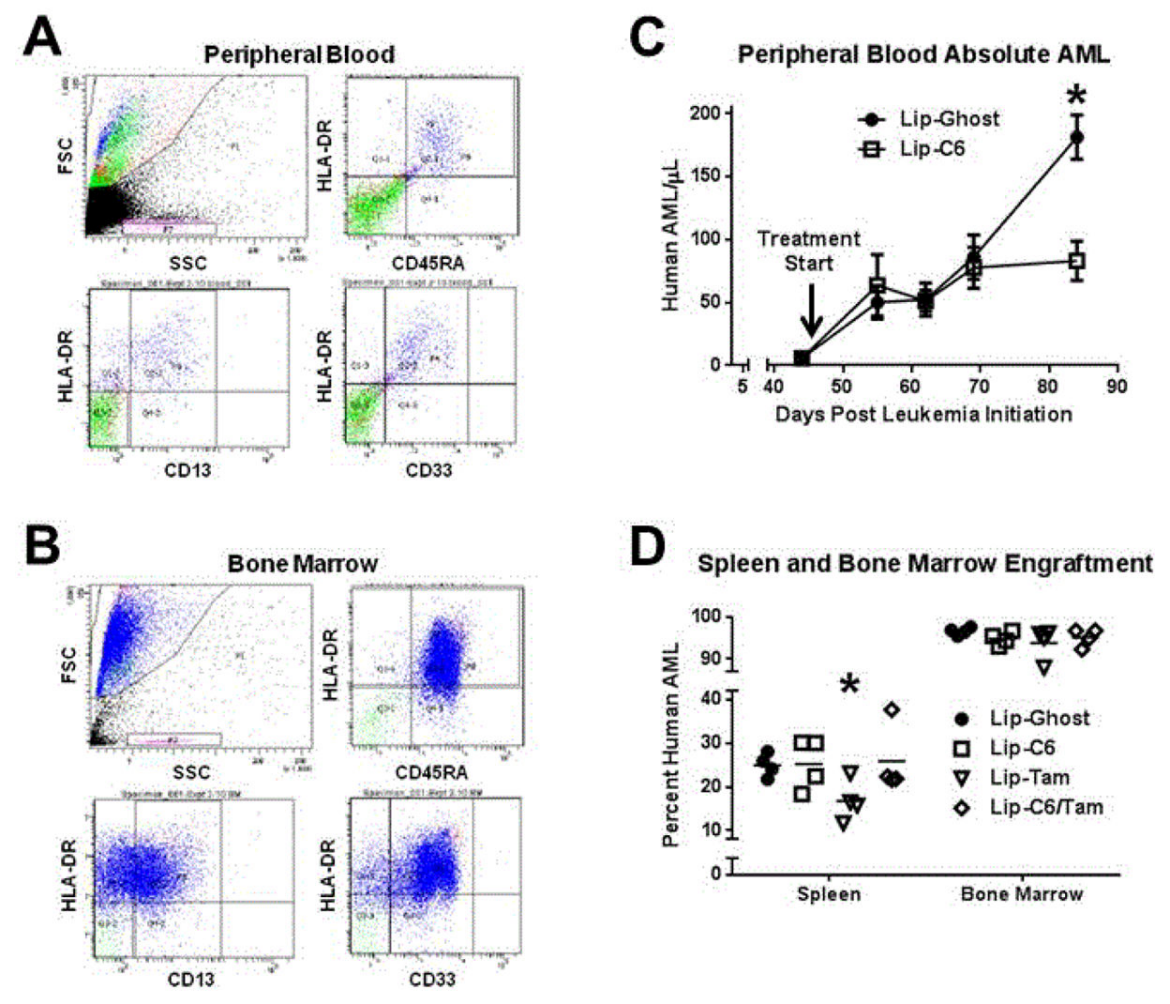
Monitoring the therapeutic efficacy of C6-ceramide and tamoxifen nanoliposomes in NSG mice engrafted with human AML. NSG mice were given sublethal total body irradiation prior to engraftment with human AML via tail vein injection. Engraftment was evaluated by flow cytometry of the peripheral blood (A), and bone marrow (B), using anti-human CD13, CD33, HLA-DR, and CD45 antibodies. (C) Treatment was initiated following engraftment confirmation, and the anti-AML efficacy of C6-ceramide nanoliposomes (Lip-C6), or control nanoliposomes (Lip-Ghost), was evaluated using NSG mice engrafted with a poor prognosis human AML sample (inv3, -7), and leukemia burden was routinely monitored by analysis of blood collected from tail vein prick. *p<0.001, 2-way ANOVA, n=4 mice per group, error bars represent standard error of the mean. (D) Human AML engraftment was assessed by flow cytometry of spleen and bone marrow preparations following necropsy at day 98. Included is a comparison with tamoxifen nanoliposomes (Lip-Tam), as well as nanoliposomes loaded with both C6-ceramide and tamoxifen (Lip-C6/Tam) (*p=0.0317, unpaired t-test comparing Lip-Ghost and Lip-Tam).

Herein, we report that AML isolated from the peripheral blood of human patients was successfully engrafted into sublethally irradiated NSG mice. Engraftment of human AML cells in NSG mice was defined by the prevalence of cells with both CD13 and CD33, or either in combination with one other human marker (HLA-DR, CD45) when monitored by flow cytometry of peripheral blood at various time points or bone marrow or spleen at necropsy (Figure 1a–d). We have also evaluated CD19 or CD3 in select cases to distinguish normal hematopoietic cells (data not shown), however co-expression of lymphoid markers with CD33 or CD13 identifies AML with multilineage dysplasia. Overall, we observed a primary engraftment efficiency of 81% (31/38 mice) for a total engraftment of 13/14 human AML cases evaluated (Table 1).

**Figure 2:**
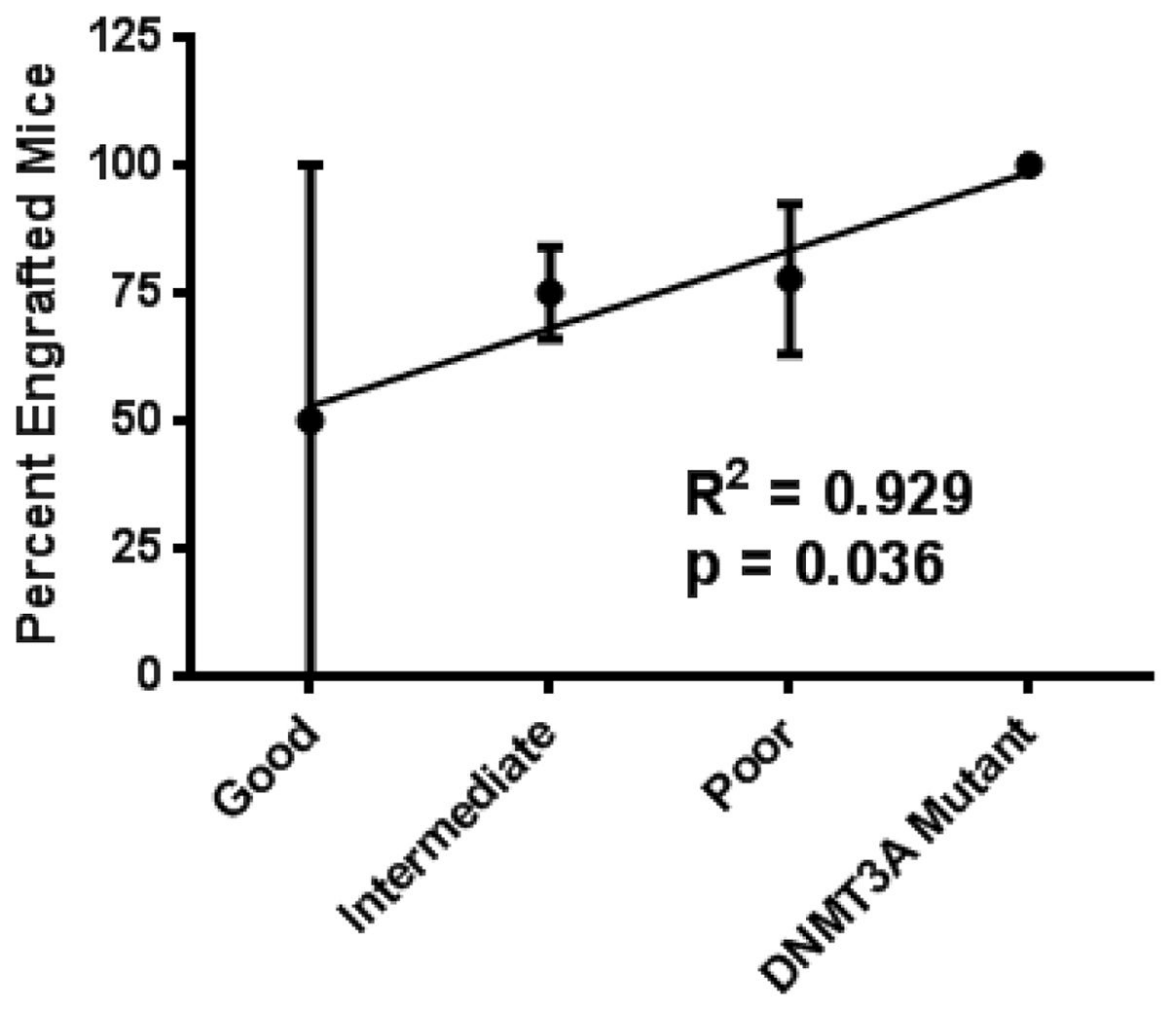
DNMT3A mutation predicts for enhanced engraftment in NSG mice. The integrated genetic risk profile, or DNMT3A mutation, was used to rank cases for linear regression analysis (1=good, 2=intermediate, 3=poor, 4=DNMT3A mutation).

We subsequently transplanted several cases from primary recipients into secondary and tertiary recipients. Subsequent engraftments were highly efficacious (near 100%), but most importantly allowed for expansion of human AML for experimental therapeutic evaluation. For primary engraftments, we confirm better engraftment of otherwise indeterminate cases which have a mutant FLT3 (88%), compared with other cases (71%). We used mutational profiling to further define the risk stratification for the human AML samples that we engrafted. This additional profiling revealed that samples with a mutation of DNMT3A engrafted at a rate of 100% (5/5 total engraftments from 3 separate specimens). We can compare the engraftment of DNMT3A mutant cases with all others based on the integrated genetic risk profile by assigning values of 1–4 for good, intermediate, poor, and DNMT3A mutants, respectively (Figure 2). In this way, linear regression analysis can be used to show that DNMT3A mutation, even beyond a poor integrated genetic risk profile, is predictive of patient cell engraftment in NSG mice. Failure of hematopoietic stem cell lineage commitment and enhanced self-renewal has been reported to be a consequence of DNMT3A mutation [22]. Therefore this mutation, which is prevalent in 22% of adult AML and is associated with increased risk of relapse [3], may play a role in maintaining and expanding the AML stem cell population.

The utility of NSG mice engrafted with primary human AML has been limited. Initially, Saito et al. used Ara-C to define a chemotherapy resistant AML stem cell population [6], and recently used the NSG model to evaluate RK-20449, a novel hematopoietic cell kinase-targeted compound identified through drug screening [11]. In another example, Herrmann et al. used the NSG model to evaluate heat shock protein inhibitors, but did so by treating patient AML in vitro prior to engraftment [12]. In our study, we evaluated the preclinical efficacy of nanoliposomal C6-ceramide and nanoliposomal tamoxifen, as well as a combinatorial ceramide/tamoxifen nanoliposomal formulation, in NSG mice engrafted with a poor prognosis primary human AML (case 329: inv3, −7). In this study we were able to readily monitor the leukemia burden in mice by analyzing tail vein blood, and noted that nanoliposomal C6-ceramide blunted the exponential growth of leukemia (Figure 1c). Additionally, at necropsy we were able to note a significant decrease in the leukemia burden in the spleen, but not the bone marrow, of mice treated systemically with nanoliposomal tamoxifen, but not other agents (Figure 1d). This study confirmed that NSG mice engrafted with human AML can be successfully used for preclinical evaluation of novel experimental therapeutics.

Various therapeutics induce ceramide generation, a bioactive sphingolipid that regulates cellular stress and death, while metabolic pathways to eliminate ceramide have gained notoriety as mechanisms of therapy resistance [13,23,24]. We have shown that tamoxifen is a potent inducer of ceramide accumulation through a mechanism preventing P-gp mediated ceramide glycosylation at the Golgi membrane [13,14]. We have demonstrated robust in vivo anticancer efficacy for nanoliposomal ceramide in preclinical models of breast cancer, pancreatic cancer, melanoma, LGL leukemia, and hepatocellular carcinoma, and recently demonstrated its combinatorial efficacy with nanoliposomal tamoxifen in an in vivo model of colorectal cancer [13,14]. Our present study revealed a modest yet significant efficacy for nanoliposomal C6-ceramide and nanoliposomal tamoxifen as anti-AML therapeutics that can reduce the blood or spleen leukemia burden, respectively. Importantly, the NSG model allowed the evaluation of a poor prognosis human AML effectively reconstituted in the murine host. It is noteworthy that the combination of nanoliposomal C6-ceramide and nanoliposomal tamoxifen did not exert therapeutic efficacy in this particular AML case (Figure 1d). This may be indicative of a further need to evaluate how the underlying biology in specific subtypes of AML relates to sphingolipid metabolism. Too many generalizations about the potential efficacy of experimental therapeutics such as nanoliposomal C6-ceramide and nanoliposomal tamoxifen have been made based on models that do not replicate the exact biology of the human disease. NSG mice engrafted with human AML offers the potential to be a more predictive preclinical model for evaluating the efficacy of these experimental therapies. Subsequent studies using nanoliposomal C6-ceramide, nanoliposomal tamoxifen, or their combination, may focus on selective targeting strategies to improve their overall therapeutic efficacy, and may focus on evaluating efficacy in molecularly specific AML subtypes. Furthermore, the expansion of human AML in the bone marrow of NSG mice can allow sufficient cell numbers to perform post-treatment analysis to evaluate changes in the sphingolipidome associated with ceramide-based therapeutics. Altogether, this work demonstrates the potential to explore in vivo drug sensitivity of molecularly defined human AML in the NSG mouse. This model is likely to prove valuable in the study of novel agents with therapeutic anti-AML activity including ceramide-based therapeutics.

## Figures and Tables

**Table 1:** 

AML Sample #	Cytogenetic Category (2010 MRC classification)	Mutations	Integrated Genetic Risk Profile	WBC (×1000/μl) at sample collection	# Mice Engrafting (> 0.5%)/Total # Mice Evaluated	Max% Primary Engraftment	Max% Secondary Engraftment (Blood)
146	Intermediate (Normal Karyotype)	FLT3-ITD, DNMT3a R882H	Poor	14.2	1/1	30 (Blood)	55
202	Intermediate (Normal Karyotype)	FLT3-ITD, IDH2 R140Q, DNMT3A R882P	Poor	58.4	2/2	13.5 (Marrow)	NA
329	Unfavorable inv3, −7)	NA	Poor	41.9	4/4	63 (Blood)	52
370	Intermediate Normal Karyotype)	FLT3-ITD, ASXL1 E1102D	Poor	61	2/2	2 (Marrow)	NA
436	Intermediate Normal Karyotype)	FLT3-ITD	Intermediate	23.6	2/2	61 (Blood)	23.9
441	Intermediate Normal Karyotype)	FLT3-ITD	Intermediate	82.7	4/4	44 (Blood)	48
448	Favorable (inv16)	NA	Good	110.6	1/2	58 (Blood)	4.9
452	Intermediate (der12, t8;12)	FLT3-ITD	Intermediate	63.5	2/4	42 (Marrow)	1.3
476	Unfavorable (Complex Karyotype)	NA	Poor	91.9	1/3	3.4 (Marrow)	NA
481	Intermediate (t9;11, +8)	FLT3 D835	Intermediate	95.75	5/6	17 (Marrow)	0
482	Intermediate (del9)	NPM1, DNMT3A R882H	Intermediate	17.03	2/2	74 (Marrow)	0
489	Intermediate (+19)	IDH2 R140Q	Intermediate	14.99	0/1	0 (Blood)	NA
541	Intermediate (Normal Karyotype)	FLT3-ITD, NPM1, IDH1 R132H	Intermediate	191. 57	3/3	1.5 (Blood)	NA
555	Intermediate (t9;11)	none	Intermediate	15.78	2/2	12.9 (Blood)	NA
